# Food-Derived Umami Peptides: Linking Candidate Discovery, Experimental Validation, and Food Applications

**DOI:** 10.3390/foods15203596

**Published:** 2026-10-10

**Authors:** Chunming Zhou, Yang Sun, Liying Zhu, Ling Jiang

**Affiliations:** 1 College of Biotechnology and Pharmaceutical Engineering, Nanjing Tech University, Nanjing 211816, China; 2 College of Food Science and Light Industry, Nanjing Tech University, Nanjing 211816, China; 3 School of Chemistry and Molecular Engineering, Nanjing Tech University, Nanjing 211816, China; 4 State Key Laboratory of Materials-Oriented Chemical Engineering, Nanjing Tech University, Nanjing 211816, China

**Keywords:** umami peptides, virtual hydrolysis, machine learning, deep learning, sensory validation, food application

## Abstract

Alternative proteins and cultivated meat increase the need for flavour strategies that maintain savoury quality with less reliance on salt and conventional seasonings. Food-derived umami peptides are promising candidates, but their practical value depends on evidence beyond sequence prediction. With advances in virtual digestion, peptidomics, and AI-assisted approaches, discovery is expanding from sensory-guided preparation toward candidate-library construction and data-driven prioritization. This review compares food-protein resources and experimental and sequence-based discovery routes, and then examines chemical and sensory validation, instrumental support, and receptor-related evidence. Structure-based calculations are considered tools for mechanistic interpretation rather than substitutes for functional assays. The review organizes these findings within a prediction–validation–application framework and assesses their relevance to food formulation, processing stability, and production feasibility. Remaining priorities include transparent taste datasets, reproducible model evaluation, and replicated food trials that connect effective dose, off-flavours, recovery yield, and cost. A data–model–experiment–food framework is proposed to guide practical umami-peptide development.

## 1. Introduction

### 1.1. Flavour Gaps, Salt-Reduction Pressure, and Opportunities for Umami Peptides

Weak umami remains a formulation problem in plant-based meat products, as illustrated by work on soy protein patties [1]. Salt and flavour enhancers can strengthen the savoury background, although sodium-containing ingredients also affect the scope for salt reduction. Food proteins offer a source of taste-active peptides: fungal studies have identified candidates with sensory activity alone or in mixtures with MSG and nucleotides [2,3]. These findings justify testing peptide preparations in alternative protein foods, but their usefulness depends on the formulation. A dose that enhances umami in a model solution still needs to maintain acceptable flavour in a product when salt or conventional seasoning is reduced. Such application claims require a defined recipe and an appropriate reduced-seasoning control.

Virtual digestion allows researchers to examine protein sequences before preparing a hydrolysate. The resulting candidates can be compared with peptidomic findings and ranked for synthesis and sensory testing. Its proposed coverage of low-abundance or transient sequences is considered in Section 3.2 within the evidence framework introduced below.

Umami peptides have been reported in meat and aquatic products, including Jinhua ham, fermented fish, and oysters [4,5,6]. Plant-protein studies include soybean-derived candidates and saltiness-enhancing peptides from rice bran [7,8]. Fermented milk and sufu provide examples from fermented foods [9,10], while edible fungus studies include *Flammulina filiformis* and *Hericium erinaceus* [11,12]. Processing by-products are also relevant: clam cooking liquid and eggshell membrane have been investigated as peptide sources [13,14]. These studies establish activity for particular materials and sequences. Protein composition determines which sequences may be released, while the accompanying flavour and processing behaviour affect how the resulting ingredient can be used. Source selection therefore influences both peptide discovery and subsequent formulation work.

### 1.2. From Empirical Discovery to Data-Driven Discovery

The conventional discovery route begins with hydrolysates or fermented samples and proceeds through fractionation, sensory-guided purification, MS identification, and synthetic confirmation. Its strength is direct contact with real substrates and real taste responses. Its weakness is scale: only a small fraction of a complex peptide mixture can be purified, synthesized, and tested. Recent workflows therefore combine wet experiments with computation. Virtual enzymatic digestion generates candidates from protein sequences and cleavage rules, as demonstrated for collagen and tomato proteins [15,16]. Peptidomics identifies sequences in experimental samples, including shiitake hydrolysates [17,18]. Prediction models then rank candidates for synthesis and sensory testing, while docking and MD investigate possible interaction patterns [19,20,21].

In this review, artificial intelligence (AI) is the broad computational category, machine learning (ML) denotes models learned from data, and deep learning (DL) is the ML subset using multilayer neural networks. Protein language model embeddings are representations that may feed different downstream predictors. Virtual enzymatic digestion is ordinarily a rule-based sequence-generation method; molecular docking and molecular dynamics are structure-based computations. These methods can be combined with AI models, but their inclusion in a workflow does not make them AI methods.

Combining sequence-based generation with experimental identification broadens the candidate space before the expensive synthesis and testing stage. The candidate-compression ratio and experimental hit rate describe different aspects of this workload; their definitions and interpretation are given in Section 4.3.

### 1.3. Current Progress, Unresolved Issues, and Review Scope

Recent studies have expanded umami peptide research in several directions. Candidate discovery has been reported for hybrid pork and eggshell membrane [14,22], while oyster and soybean studies illustrate aquatic and plant-protein sources [6,7]. Reviews have considered livestock and poultry by-products [23] and the wider range of food-derived umami peptides [24]. These bodies of work differ in their starting materials and validation endpoints. Virtual hydrolysis, peptidomics, molecular docking, molecular dynamics, and machine learning have been incorporated into screening workflows, but several problems remain unresolved. Public datasets are still small and heterogeneous, external validation is limited, and many predicted sequences have not been tested in real food matrices. Detected, model-predicted, synthetic, receptor-associated, and food-matrix-tested peptides consequently provide different kinds of evidence.

This review therefore follows the route from food-protein resources and candidate-library construction to virtual hydrolysis, peptidomics, AI-assisted prioritization, synthetic validation, sensory and electronic-tongue evaluation, T1R1/T1R3-related receptor evidence, molecular docking and dynamics, and food-system application. Particular emphasis is placed on how virtual hydrolysis expands the sequence-discovery space beyond empirically detected peptides and how predicted candidates should be verified through synthetic peptides, receptor-level evidence, sensory testing, and food-matrix experiments. The data–model–experiment–food framework used here follows these candidates through discovery and testing to examine how well their reported activity supports food use.

**Figure 1:**
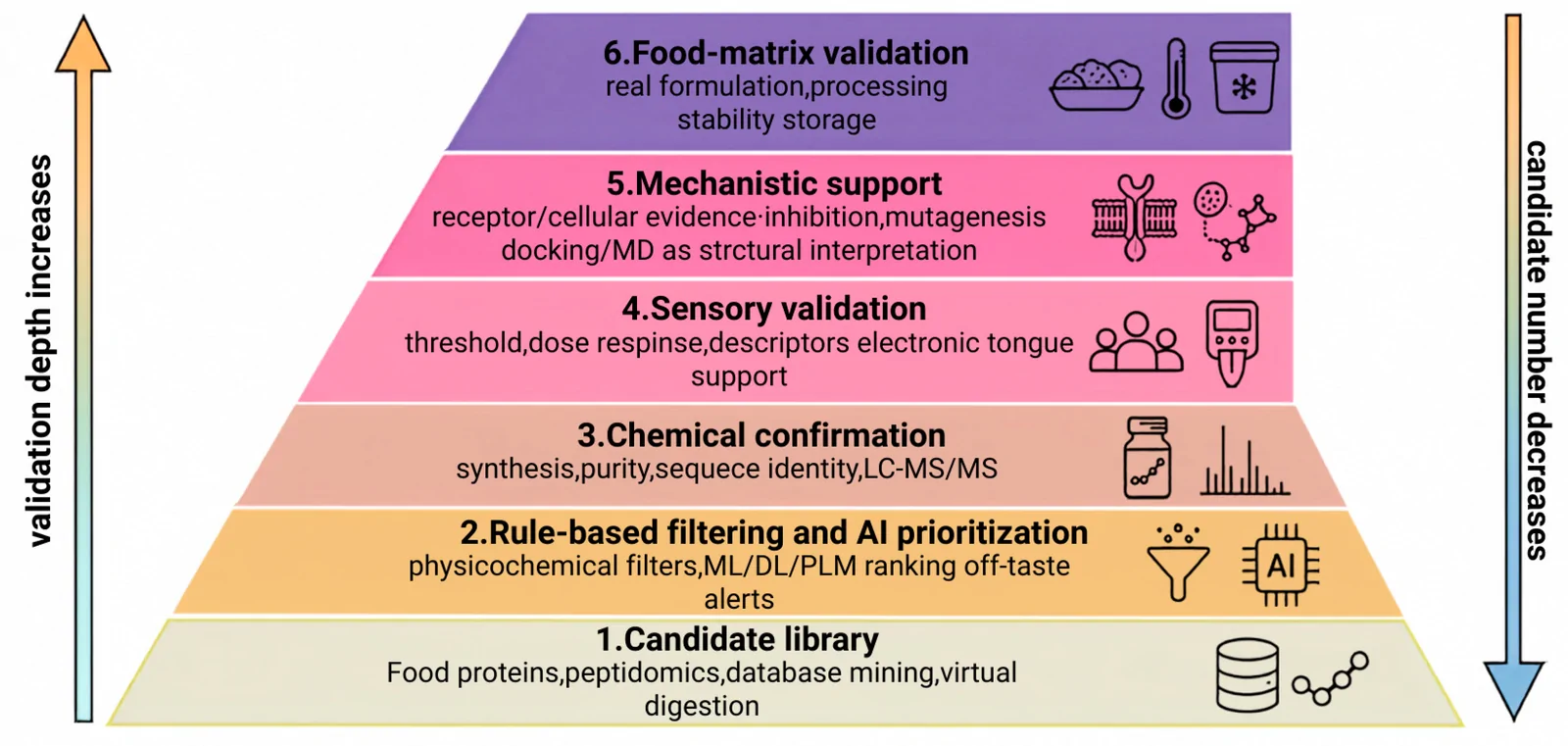
Evidence-transition framework for umami-peptide discovery. Candidate selection is linked schematically to increasing validation depth, from library construction to food-matrix testing. Docking and MD provide structural interpretation rather than functional receptor evidence.

Previous reviews have summarized food-derived umami peptides and methods for their screening and evaluation [24,25]. Processing routes and computational predictors have also been reviewed [26,27]. Here, we examine what the evidence establishes at each stage of development. Figure 1 organizes candidate generation, prioritization, chemical confirmation, sensory validation, mechanistic support, and food-matrix testing. Three interpretive boundaries apply throughout this review: virtual cleavage does not establish release during food processing; docking or MD does not establish functional receptor activation; and activity in a model solution does not establish efficacy in food. These boundaries are stated here and applied to specific assays and formulations in Section 3, Section 4, Section 5 and Section 6.

### 1.4. Literature Identification and Selection

This is a critical narrative review, not a systematic review or a scoping review. Literature identification combined PubMed and the Semantic Scholar Academic Graph API.

The topic block comprised “umami peptide”, “umami peptides”, “umami-enhancing peptide”, and “umami-enhancing peptides” in Title/Abstract, combined by AND with screening, identification, discovery, peptidomics, sensory evaluation, virtual screening, machine learning, deep learning, docking, molecular dynamics, T1R1, or T1R3. The main Semantic Scholar query was “umami peptides virtual screening enzymatic hydrolysis machine learning molecular docking sensory evaluation”. Application queries combined umami peptide with food application, salt reduction, MSG, food matrix, plant-based meat, stability, thermal processing, storage, by-product hydrolysate, and flavour enhancer; separate queries covered kokumi, saltiness enhancement, and yeast extract.

The working literature pool was deduplicated using DOI where available and title/year matching as a fallback. Title/abstract screening prioritized food-derived umami or umami-enhancing peptides, candidate-generation and prediction methods, receptor-related evidence, sensory validation, and food application. Related salty/kokumi peptides and general receptor studies were retained only for an explicitly relevant comparative or mechanistic purpose. Records unrelated to these questions and computational studies without a relevant food/taste link were excluded. Full-text evidence was checked before quantitative claims were incorporated. Reviews supplied context; primary studies supported specific measurements. Selection was purposive rather than an exhaustive synthesis, with possible bias from database coverage, keyword choices, and availability of interpretable full-text evidence.

## 2. Food-Protein Resources and Virtual Generation of Umami-Peptide Candidates

**Figure 2:**
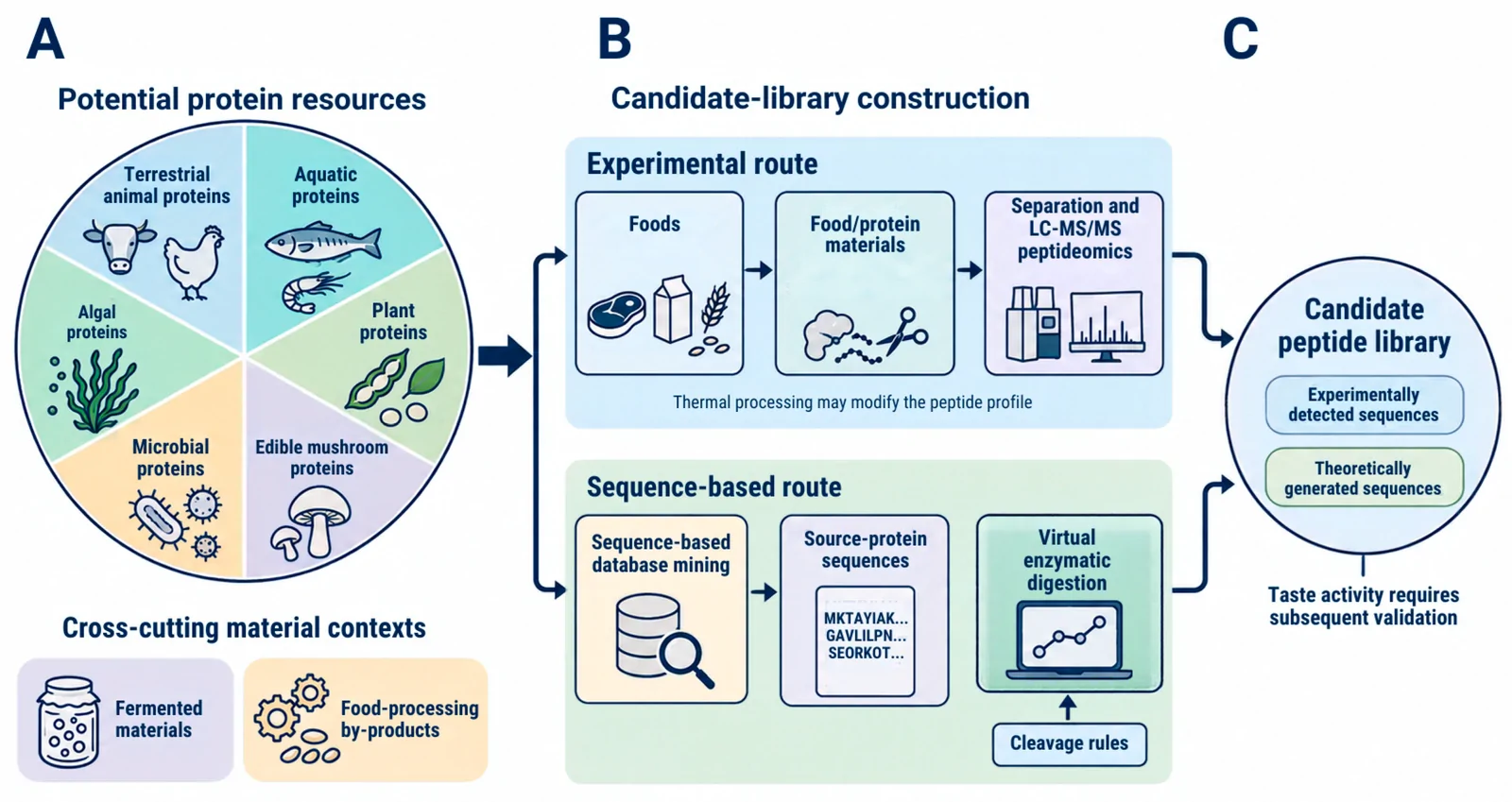
Protein resources and routes for candidate-library construction. (**A**) Potential protein resources and cross-cutting material contexts. (**B**) Experimental and sequence-based routes for candidate-library construction. (**C**) Candidate peptide library comprising experimentally detected and theoretically generated sequences. Source categories and discovery routes are not paired one-to-one. Candidate generation is distinct from taste validation.

Food-protein resources determine the starting sequence space, background flavour, processing constraints, and intended application. The source categories used here distinguish animal materials, plant materials, edible fungi, microbial biomass such as yeast, and algal/cyanobacterial biomass. Aquatic materials are animal sources, while fermentation and by-product recovery describe processing or provenance rather than mutually exclusive biological sources. Figure 2 presents source and discovery-route dimensions; a source may enter several routes, and a route may be applied to several sources. Table 1 compares representative candidates and identifies the material and endpoint actually tested.

### 2.1. Animal Sources

Meat, bone, poultry, and aquatic products have been investigated as sources of taste-active peptides. Traditional meat products contain reported umami and kokumi peptides within a cooked meat or broth-like flavour background [35]. In Jinhua ham, screening of more than 2000 peptide fragments led to 10 retained umami peptides with thresholds of 0.1109–0.2634 mg/mL [4]. Porcine bone peptides were examined using prediction tools, synthesis, and sensory evaluation [20]; preserved egg yolk [36] and bovine bone soup [37] provide further animal-derived examples. Aquatic studies have investigated crab peptides [38], oyster peptides [6], and peptides from clam cooking liquid [13]. Fermented milk and fermented fish extend this evidence to processed animal-derived systems [5,9]. These studies identify candidates within realistic flavour backgrounds, although activity of an isolated sequence and its contribution to the original food remain separate questions.

### 2.2. Plant Sources

Plant proteins are major substrates for plant-based meat, low-salt seasoning, and clean-label flavour systems. Virtual digestion of tomato proteins combined with machine learning screening identified AGPNY, which showed an umami threshold of 0.0625 mg/mL. Docking and molecular dynamics suggested possible interactions with T1R1/T1R3 and GRM1, complementing the sensory and electronic-tongue measurements [16]. These endpoints are interpreted using the distinctions in Figure 1. Soybean-derived virtual peptides and rice bran peptides have also been screened for umami or saltiness-enhancing properties, respectively [7,8]. Plant proteins are attractive because of their availability, but beany notes, bitterness, astringency, and protein-network effects can complicate formulation. Candidate discovery should therefore be evaluated together with off-flavour control and matrix performance.

### 2.3. Fungal, Microbial, Algal, and Fermented-Food Sources

Edible fungi provide peptide candidates within glutamate- and nucleotide-rich flavour backgrounds. Studies on *Russula vinosa*, truffle, and shiitake combine sensory evaluation with peptidomics or computational screening [2,3,17]. Related studies on *Hericium erinaceus*, *Flammulina filiformis*, and *Hypsizygus marmoreus* use comparable sensory and computational strategies [11,12,39]. Fermented foods are classified by both substrate and process: soy sauce, sufu, and broad-bean paste are fermented plant systems [10,40,41], whereas lager and non-alcoholic beer provide beverage matrices [28,42]. Fermentation can change the substrate peptide pool, but detection in a fermented product alone does not identify whether a sequence originated from food proteins or microbial biomass. Microbial protein sources therefore require their own source-specific evidence.

Yeast extract provides microbial-biomass evidence distinct from fermentation of plant or animal substrates. Wang et al. reported DWTDDVEAR among sensory-active candidates after fractionation, MS identification, and virtual screening [32]. Gao et al. identified FE, YDQ, FQEY, and SPFSQ and confirmed their taste using synthesized peptides [33]. These findings justify a microbial-source category, while Table 1 records the validation endpoint rather than inferring food efficacy from extract origin.

Algal/cyanobacterial biomass requires a different attribution boundary. In *Spirulina* acid hydrolysate, an umami fraction contained glutamyl di- and tripeptides together with free glutamate and succinic acid [34]. This is fraction-level evidence, not validation of an isolated peptide. *Spirulina* is a cyanobacterium included here as an algal-food resource, not a eukaryotic alga; its findings do not establish equivalent sequences or performance in other algal proteins. Source-specific purification and taste tests are needed before generalizing between these biomasses.

Evidence categories follow Figure 1: E1, computational candidate; E2, chemically confirmed sequence; E3, sensory activity in a model system; E4, receptor/cellular mechanistic support; E5, sensory performance tested in a food matrix. Categories identify the highest endpoint summarized here, not mandatory consecutive prerequisites. S denotes explicitly reported synthetic-peptide testing; I denotes electronic-tongue or other instrumental taste support. Docking/MD alone does not qualify as E4. NR, not reported; UAEH, ultrasound-assisted enzymatic hydrolysis; GAT, graph attention network; PTM, post-translational modification; MM-GBSA, molecular mechanics/generalized Born surface area.

## 3. Methods for Obtaining Umami Peptides

### 3.1. Conventional Preparation Routes

Experimental preparation establishes which peptides are obtained from a food protein under defined processing conditions. Enzymatic hydrolysis produces mixtures for subsequent screening, as illustrated by Jinhua ham, oyster, and *Flammulina filiformis* studies [4,6,11]. Fermented fish and sufu provide examples of peptides investigated in fermented materials [5,10]. Thermal reactions can further transform hydrolysates into complex Maillard flavour preparations [43]. Membrane separation and chromatography simplify peptide mixtures, and MS identifies sequences in selected fractions [25]. The peptide profile varies with the protease and processing conditions. Changes in hydrolysis time, pH, or temperature can alter cleavage and the resulting distribution of peptide lengths, charges, and hydrophobic residues, with consequences for taste. These preparation routes must be distinguished when interpreting the origin of an active sequence or mixture.

In sensory-guided fractionation, the taste of each fraction determines which material proceeds to further purification. A hydrolysate can be separated into fractions, evaluated by sensory panels or taste-sensing instruments, identified by MS, and then confirmed with synthetic peptides. Its practical limitation is the low number of fractions and sequences that can be purified and tasted in depth. Abundant peptides are more likely to be carried forward than low-abundance peptides with high activity. Peptidomics alone can produce many sequences, but sequence detection does not establish the main contributors to umami. Mechanistic studies need highly purified peptides, whereas food applications often depend on the combined contribution of peptides, glutamate, nucleotides, organic acids, salts, and Maillard products in partially purified hydrolysates.

### 3.2. Candidate Expansion Through Sequence Databases and Virtual Hydrolysis

Virtual enzymatic digestion begins with a protein sequence and a defined set of cleavage rules. Public databases provide sequences that can be examined before hydrolysates are prepared. Studies on collagen and tomato proteins constructed theoretical peptide libraries in this way [15,16], and a soybean study screened candidates obtained by virtual digestion [7]. Experimental peptidomics supplies a complementary starting point: work on shiitake, *Hericium erinaceus*, and *Flammulina filiformis* used hydrolysates and MS identification to obtain candidates for computational screening [11,12,18]. Both routes can guide synthesis, but their starting evidence differs, as defined in Figure 1.

In hydrolysis-first discovery, enzyme specificity, fractionation, MS coverage, and sensory selection successively restrict the accessible peptide pool. Virtual digestion instead enumerates sequences permitted by the chosen cleavage rules before physicochemical filtering and model ranking. This difference concerns candidate coverage rather than demonstrated peptide recovery. A matched comparison would need to quantify the overlap between theoretical and detected libraries under the same substrate and processing conditions.

Reported studies provide sensory-active candidates from both routes. AGPNY from virtual digestion and screening of tomato proteins had an umami threshold of 0.0625 mg/mL [16]. Studies starting from experimental fungal hydrolysates reported thresholds of 0.05–0.15 mmol/L for *Flammulina filiformis* peptides [11] and 0.07–0.23 mmol/L for *Hericium erinaceus* peptides [12]. The retained Jinhua ham candidates had thresholds of 0.1109–0.2634 mg/mL [4]. These findings do not constitute a controlled comparison of candidate coverage. Potency comparisons also require compatible threshold definitions, units, matrices, and sensory protocols.

### 3.3. Candidate Generation by Virtual Hydrolysis and Peptidomics

Virtual hydrolysis and peptidomics supply the theoretical and experimentally detected candidates illustrated in Figure 2. In the Jinhua ham study, more than 2000 fragments were screened, 13 candidates underwent taste evaluation, and 10 were retained after three candidates with anomalous thresholds were excluded [4]. A shiitake study moved from 1719 identified peptides to 722 model-positive peptides and four validated peptides [18]. Fermented sausage research used 150,000 nonredundant, metagenome-derived candidate sequences for prediction and ranking [29]. Library size, the number synthesized, and the final validated count describe different stages and should be reported separately.

The categories in Figure 2 should be read at distinct levels. Biological source identifies the protein resource; hydrolysis, fermentation, thermal treatment, and simulated digestion describe experimental release or transformation; LC-MS/MS peptidomics identifies sequences in samples; database retrieval and virtual enzymatic digestion generate sequence-based candidates. Fermented foods and by-products can originate from several biological sources and are not additional taxonomic groups. These routes need not form one fixed sequence: experimental identification and theoretical enumeration can proceed in parallel before prioritization.

High-priority candidates can be synthesized for sequence-level testing and then examined in hydrolysates or food matrices. Section 5 describes the chemical, sensory, instrumental, and receptor-related endpoints. Table 2 compares the functions and limitations of wet enzymatic hydrolysis and virtual enzymatic digestion.

### 3.4. Data Features, Labels, and Negative Samples

Taste labels from separate studies may describe different experimental outcomes. Published sequences, taste-peptide databases, and curated literature provide much of the training evidence [27,44]. Task definitions also vary. Umami_YYDS classifies umami and bitter peptides [45], whereas other predictors compile umami and non-umami records [44,46]. A bitter sequence and an untested sequence are therefore not interchangeable negative examples. Peptide purity, test concentration, and the sensory protocol should accompany each activity label so that records can be interpreted in context. Activity established with a synthetic peptide and a trained panel carries different support from a prediction or an instrumental response alone. An untested sequence should not receive a negative taste label solely because no umami activity has been reported.

Dataset size is an incomplete indicator of reliability. A large library may improve coverage, but model performance can still be inflated when homologous peptides are split across training and test sets, when negative samples are untested peptides, or when positive labels come from heterogeneous taste assays. Studies that report redundancy filtering, train/test partitioning, independent testing, and experimental follow-up are more informative than studies that report only cross-validation accuracy. The same point applies to virtual-hydrolysis outputs: a long candidate list is useful only when the downstream filtering logic and validation endpoint are clear.

Feature choice shapes how candidate peptides are ranked. Conventional descriptors—amino-acid composition, dipeptide and tripeptide composition, peptide length, molecular weight, hydrophobicity, charge, isoelectric point, and acidic residue content—remain useful for small datasets and interpretable screening. Sequence embeddings retain information about residue order and neighbouring amino acids that composition-based descriptors omit. Whether this improves screening needs to be tested on independent sequences and followed through to experimental confirmation. Label definitions and sequence overlap between datasets remain important when interpreting any gain in predictive performance.

## 4. Machine Learning and Deep Learning Screening

### 4.1. Conventional Machine Learning Models

**Figure 3:**
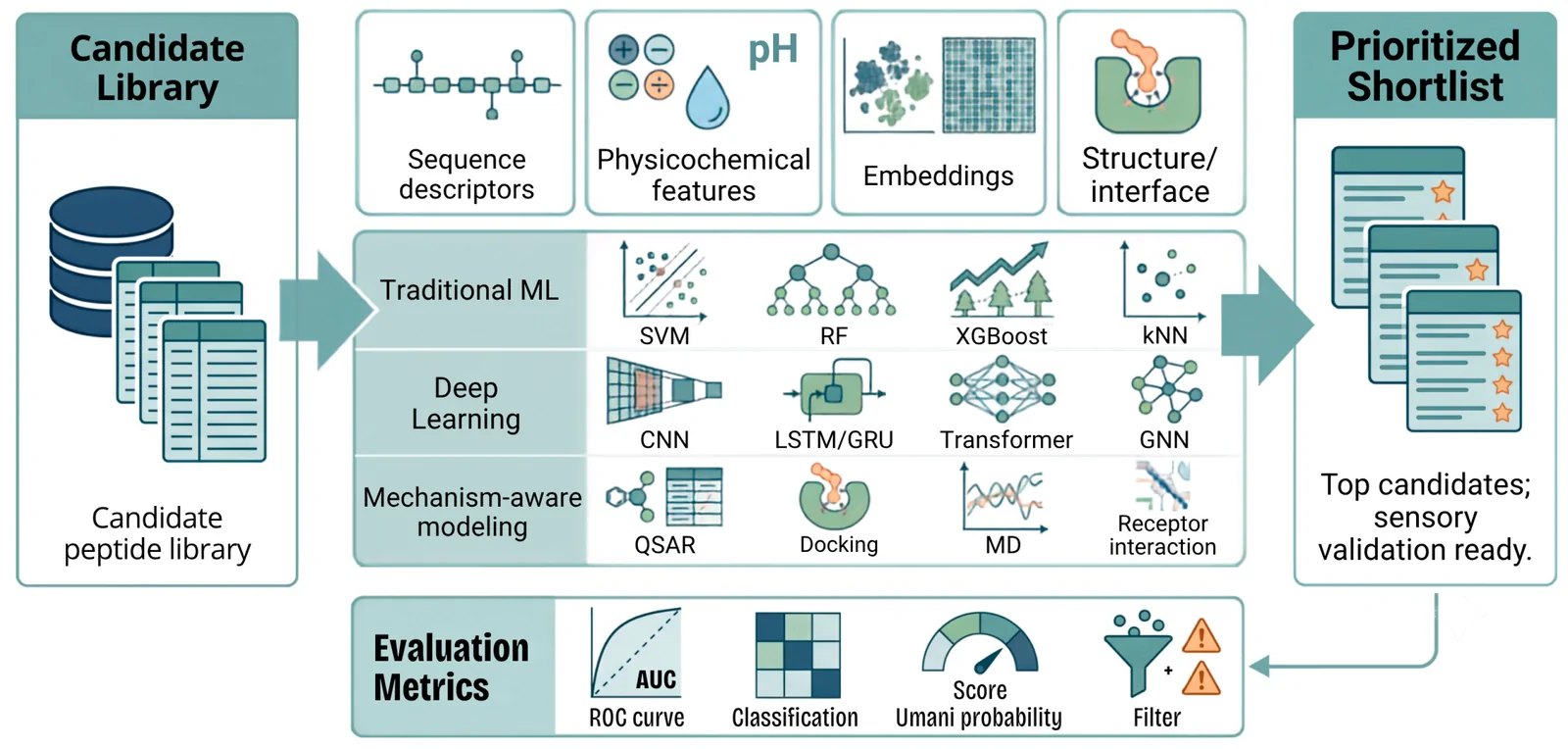
Computational prioritization of candidate peptides using sequence, physicochemical, embedding, and structure-related information. Model assessment and experimental screening outcomes are distinguished in Section 4.3.

Descriptor-based methods provide relatively compact, interpretable baselines when labelled taste data are scarce [21,47]. Scoring cards expose residue and dipeptide contributions [48], whereas feature representation predictors combine complementary descriptors [49]. The iUmami-SCM and UMPred-FRL studies use the same UMP-TR/UMP-IND split, so their scores describe performance within a shared curated benchmark, not transfer to a new food source [48,49]. Bitter peptide negatives also frame a narrower discrimination task than distinguishing umami from all possible non-umami sequences. These models can prioritize synthesis, as illustrated by porcine bone work [20], but their usefulness should be judged by prospective candidate testing as well as classification metrics. Small model size and interpretability do not remove label uncertainty, similarity leakage, or partition selection bias. Figure 3 summarizes the sequence, physicochemical, embedding, and structure-related information used for computational prioritization.

### 4.2. Deep Learning and Protein Language Models

Sequence representations capture residue context beyond hand-crafted composition features. However, architecture comparisons are entangled with training data and evaluation design. iUP-BERT and iUmami-DRLF reuse the UMP442 benchmark employed by earlier descriptor-based predictors [50,51]; their representation changes do not constitute independent external validation. Umami-BERT uses a different dataset and class ratios, while UmamiMeta combines larger, heterogeneous taste records and evaluates length-dependent performance [44,52]. These differences affect the learning task and preclude interpreting higher reported accuracy as an architectural advantage across studies. Pretraining can supply useful sequence features, but limited and inconsistent taste labels still constrain the downstream predictor.

Ensemble learning is a separate design choice from sequence representation. TastePeptides-Meta contains a database, an umami/bitter GBDT classifier, and prediction software [45]; Umami-gcForest combines sequence-derived features with deep-forest learning [53]. Their strengths are complementary features and flexible downstream classification, while limitations include negative-label provenance and the selection of configurations using holdout results. Experimental follow-up also differs: Umami-Transformer synthesized and taste-tested selected short peptides [19], whereas the fermented-sausage workflow tested three synthetic peptides in water/MSG rather than reconstituting their effect in sausage [29]. Classification-only benchmarks, positive-only verification sets, selected-candidate taste tests, and food trials answer different questions. Table 3 separates these endpoints and records the reporting limitations needed to interpret each result.

Table 3 separates predictive metrics from experimental follow-up and documents the evaluation setting for each reported value. An internal holdout drawn from the same curated pool is not equivalent to an independently collected external dataset, even when the source calls it an independent test. Reuse of UMP-IND across studies and the use of holdout scores for configuration selection further limit independence. Calibration, cross-validation, and holdout results therefore should not be ranked as a common benchmark. Sequence deduplication does not establish homology-separated splitting; related sequences require an explicit similarity threshold and grouping rule. Resampling and feature selection should occur within training partitions, leaving validation and test data untouched. Table 3 records uncertain resampling order rather than assuming that leakage was excluded. For the first four models, Table 3 presents study-specific methods and reported performance alongside their shared data conditions and model-specific reporting differences. These entries should not be counted as four independent datasets or external validations.

### 4.3. From Model Prediction to Experimental Prioritization

Within the framework in Figure 1, prediction denotes computational prioritization of a sequence; validation denotes confirmation against a specified chemical, sensory, instrumental, or receptor endpoint; food functionality denotes a measured effect at a stated dose in a defined formulation. A candidate can satisfy one endpoint without satisfying the others, and functional receptor evidence is not inferred from docking. Prediction studies and sequence-modification work can guide matched synthesis and sensory comparisons [46,54]. Screening efficiency should be reported separately from classification performance: the candidate-compression ratio is CR = Nlibrary/Nsynthesized and the experimental hit rate is HR = Nvalidated/Nsynthesized, with the validation criterion stated explicitly. HR based on a few selected peptides is imprecise and dose-dependent. Shiitake work narrowed 1719 identified peptides to four validated candidates [18], Jinhua ham work retained 10 after testing 13 candidates from more than 2000 fragments [4], and fermented-sausage research ranked 150,000 metagenome-derived candidates [29]. These counts describe workload; food-functionality claims require the formulation tests discussed in Section 6.

## 5. Experimental Validation and Mechanistic Evidence

The evidence-transition framework in Figure 1 separates chemical identity, sensory activity, instrumental support, receptor-related experiments, and food-matrix performance. This section examines the endpoints and reproducibility requirements at each stage.

### 5.1. Chemical Confirmation of Candidate Peptides

Chemical confirmation is the first step after computational or peptidomic prioritization. A predicted sequence should be synthesized or isolated, checked for purity, and matched to its expected mass or fragmentation pattern before its taste activity is interpreted. Solid-phase peptide synthesis is widely used because it allows a single sequence to be tested without the background of amino acids, salts, nucleotides, Maillard products, or coexisting peptides. Studies on Jinhua ham, shiitake, and tomato protein candidates show why this step matters: virtual hydrolysis or peptidomics can nominate many sequences, but only chemically confirmed candidates can be assigned sequence-level activity [4,16,18].

Chemical confirmation limits over-attribution in hydrolysates. Synthesis and reconstitution allow a candidate to be separated from its background, tested alone, and compared with the original fraction. Reports should document peptide purity, identity checks, stock preparation, and the contribution of residual salts or other impurities to the assay.

### 5.2. Sensory Validation and Threshold Interpretation

Sensory testing characterizes a chemically confirmed peptide over a concentration range. A detection threshold records discrimination from a blank; a recognition threshold records the identification of taste quality. Dose–response measurements and sensory descriptors characterize responses above threshold. Screening has yielded candidates from tomato protein, edible fungi, and Jinhua ham with measurable sensory activity [4,11,16]. Thresholds should be interpreted alongside the test protocol rather than used as universal potency rankings.

Reports should specify the threshold definition, concentration unit, reference tastant, pH, salt content, panel size and training, sample coding, presentation order, and replicate measurements. Converting mg/mL to mmol/L requires the peptide molecular weight. Comparisons under a common protocol are more informative than rankings assembled across studies; the formulation endpoint is addressed in Section 6.

### 5.3. Electronic Tongue as Instrumental Support

Electronic-tongue measurements can support sensory interpretation by showing changes in umami, saltiness, bitterness, aftertaste, or overall taste fingerprints. They are useful for screening fractions, comparing hydrolysates, and monitoring taste changes during processing. However, electronic-tongue output is not receptor-level evidence. It should be presented as instrumental taste support that complements trained-panel or consumer evaluation. When electronic-tongue results and sensory data disagree, sensory evidence and matrix-specific validation should guide the final interpretation.

For food applications, electronic-tongue measurements need to be interpreted alongside sample composition and sensory results. A comparison across food categories examined the relationships among perceived umami, equivalent umami concentration, and electronic-tongue responses, illustrating the need for matrix-specific interpretation [55]. Jinhua ham and shiitake studies combined MS-based candidate identification with synthesis, electronic-tongue testing, and sensory evaluation [4,18]. This combination helps connect detected sequences with taste activity. It does not by itself quantify peptide retention during processing. To investigate an effect of heating, pH adjustment, fermentation, or storage, measurements of the target peptides before and after treatment should accompany instrumental and sensory results. A changed taste signal alone cannot identify which molecules caused the change.

### 5.4. Receptor-Related Experimental Evidence

Mechanistic claims require evidence closer to the receptor or cellular response. A taste-associated metabotropic glutamate receptor variant has been investigated in glutamate signalling [56]. Foundational studies established T1R1/T1R3 as a receptor system involved in mammalian and human umami perception [57,58,59]. For peptides, the interpretation depends on the assay. Phage-display screening targeting the T1R1 Venus flytrap domain has identified candidates with receptor-association evidence [60]. Such binding-oriented findings do not establish functional activation of the intact receptor. Cellular signalling assays examine functional responses, while inhibition or mutagenesis can test the involvement of a proposed receptor or contact residue. These endpoints should be distinguished from peptide detection, sensory activity, and computational binding predictions when assessing how much of a proposed mechanism has been demonstrated.

Binding assays measure target association, whereas functional involvement is more directly tested by a cellular response that changes after receptor inhibition or mutation. Receptor species, expression system, assay controls, and concentration range determine how such findings can be interpreted. Where receptor-level and sensory results differ, reporting both is more informative than selecting the endpoint that best supports the proposed mechanism.

### 5.5. Molecular Docking and Molecular Dynamics

Docking generates a proposed peptide–receptor pose, and molecular dynamics follows the simulated complex to examine contacts and conformational changes. Shrimp peptide studies used these methods to investigate possible interactions [61], while computational work on umami dipeptides examined receptor recognition and molecular modification [62]. These calculations suggest residues for mutation or variants for synthesis. Reproducibility requires disclosure of the receptor model, protonation and solvation assumptions, docking settings, force field, simulation length, and replicate trajectories. Interpretation follows the evidence boundaries in Figure 1.

Docking and MD can connect ranking with experimentally testable hypotheses and can be revisited after assays to explain discordant results. A useful report links the predicted contacts to the tested sequence, assay concentration, and available receptor measurements, allowing an independent reader to assess how much of the proposed interaction has been tested.

## 6. Food-System Application, Flavour Modulation, and Translational Value

**Figure 4:**
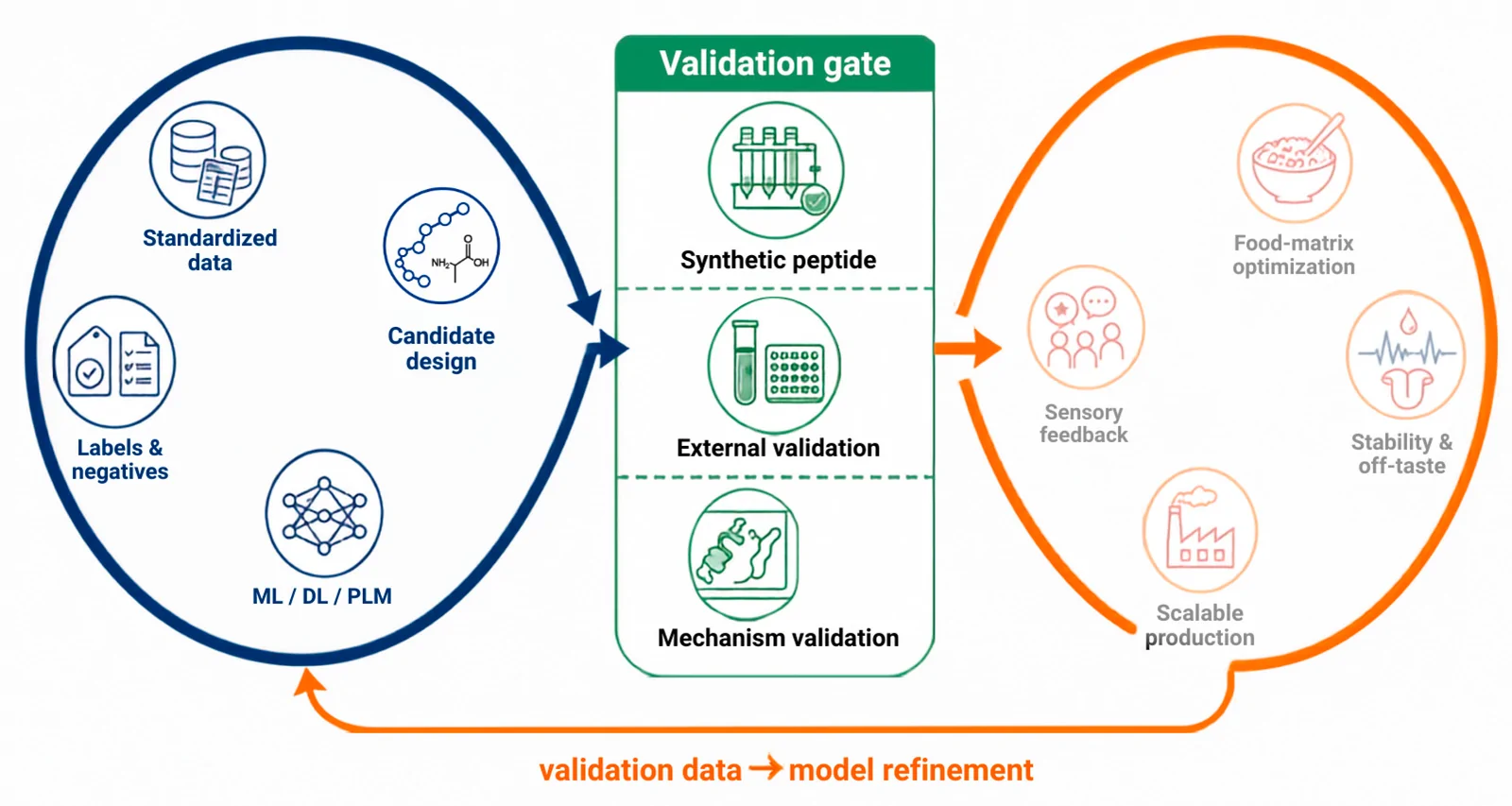
Translational pathway linking data and model development, experimental validation, food-matrix optimization, and feedback to model refinement. Product-specific tests address sensory performance, stability, off-taste, and production feasibility.

Food application tests whether a preparation improves a defined product at a usable dose. The material actually tested determines attribution: chemically confirmed isolated peptides support sequence-level effects, while hydrolysates, Maillard products, fermented bases, and compound seasonings support ingredient-level effects unless reconstitution establishes an individual contribution. Figure 4 links candidate selection to formulation and feedback. Comparisons require full-seasoning references, reduced-seasoning controls where relevant, and the sensory and processing conditions of the intended product.

### 6.1. Umami Enhancement and Seasoning-System Synergy

At 1% addition in an umami model chicken soup, a γ-glutamyl-transpeptidase-modified *Pleurotus geesteranus* hydrolysate increased sensory umami and saltiness by 35.56% and 57.69%, respectively, relative to the corresponding model without the preparation [63]. *Agrocybe aegerita* peptides tested at 5 mg/mL gave umami intensities equivalent to 4.457–5.240 mg/mL MSG, with DDL producing the strongest response [64]. Truffle peptides altered umami perception in MSG or IMP mixtures [3]. Together, these studies show that the seasoning background is an important test variable: whole-preparation enhancement and isolated-peptide potency are complementary outcomes, not interchangeable formulation doses.

### 6.2. Flavour Improvement and Off-Flavour Balancing in Food Matrices

Glutaminase treatment of wheat-gluten hydrolysate for 180 min increased umami score from 1.62 to 4.27 and reduced bitterness from 1.33 to 0.65 [65]. Golden pomfret hydrolysates gave the highest sensory score around reaction pH 7 during Maillard flavour development [43]. These findings favour evaluating umami together with bitterness and aftertaste rather than optimizing umami intensity alone. Salt, fat, proteins, and other recipe components should be included in subsequent formulation comparisons.

Formulation reports should document dose, recipe, processing conditions, sensory design, and replicate batches. Composition and target-peptide measurements can then help explain a benefit established by sensory testing.

### 6.3. Plant-Based Foods, Fermented Foods, and Product Scenarios

In soy-based textured-protein patties, protease with protein-glutaminase gave higher sensory umami, lower bitterness, and greater overall acceptability than protease with glutaminase [1]. Pea-protein-derived Maillard intermediates at 0.1% (*w*/*w*) maintained saltiness in 0.4% NaCl relative to a 0.5% NaCl reference, a 20% reduction tested in solution [66]. In a separate patty study, 1% heme-protein-hydrolysate/xylose Maillard products increased hardness from 12.54 to 15.95 N and improved umami while retaining heme-like flavour [67]. The patty findings connect taste with texture and acceptance; the pea-protein result instead motivates food trials. Increased hardness is beneficial only when compatible with the target product texture.

Hybrid pork research linked peptidomics, machine learning, and molecular simulation to peptides in a meat matrix [22]. Across these product scenarios, the development question shifts from which sequence tastes umami to which preparation corrects a specific product weakness. Bitterness, beany notes, cooked-flavour balance, texture, and consumer acceptance should determine the formulation endpoints.

### 6.4. Natural Umami Bases and Compound Seasoning Systems

Protein-rich by-products and low-value processing streams are relevant when they can be converted into natural umami bases, hydrolysates, fermented bases, Maillard reaction products, seasoning powders, or compound flavour ingredients. Shrimp-head protein hydrolysate has been formulated as a flavour enhancer, and a formula containing 40% shrimp-head hydrolysate showed sensory characteristics close to commercial seasoning [68]. Pigeon-meat hydrolysate prepared by flavour protease showed a degree of hydrolysis of 15.87%, 0.61 mg/mL sweet amino acids, and 367.45 μg/mL nucleotides, providing compositional and process indicators for natural seasoning development [69].

Shiitake work linked enzymatic hydrolysis to peptide profiles using peptidomics and in silico screening [17]. For seasoning bases, a useful specification must capture peptides together with the free amino-acid, nucleotide, and thermal-reaction background. Batch consistency, bitterness, solubility, and processing tolerance are therefore more informative than a single isolated peptide threshold.

### 6.5. Salt Reduction, MSG Reduction, and Clean-Label Applications

Salt reduction and MSG reduction are distinct formulation questions. Maintaining salty–umami perception when NaCl is reduced does not establish the amount of MSG that can be replaced. In a model solution, 0.01% synthetic GDNVGF enhanced saltiness by 17% in 70 mmol/L NaCl [70]. Cantonese-sausage research compared NaCl-replacement formulations using KCl and calcium ascorbate, with spent-hen meat hydrolysate included in two of the three replacement groups. The reported sodium reduction of approximately 40–70% therefore relates to the combined replacement schemes, rather than to hydrolysate alone [71]. A scoping review and a perspective discuss broader use of umami tastants for sodium reduction [72,73], while a review of salty peptides addresses a related but distinct sensory endpoint [74]. Studies of chicken-hydrolysate-derived Maillard products, peanut peptides, and *Pleurotus eryngii* peptides provide further saltiness-related evidence [75,76,77]. The tested material and matrix should accompany each result before its relevance to a reduced-salt food is assessed.

Clean label is a consumer-facing description, not a uniform regulatory category. Source, production process, ingredient composition, dose, and safety need to be disclosed.

### 6.6. Processing, Storage, and Digestive Stability

*Volvariella volvacea* peptide-derived Maillard products prepared at 130 °C had a saltiness index of approximately 1.40 (40% enhancement) and an electronic-tongue umami response of 18.85 ± 0.03 [78]. This characterizes the reaction product, not the retention of the starting peptide. A seasoning study tested responses under varied pH, salt, and sugar conditions, including sensory tests in seasonings [79]. These results support measuring performance after processing, while before/after target-peptide measurements are needed to distinguish retention from the formation of new taste-active products. Digestion tests address a separate exposure or safety question.

Stability tests should reproduce the stresses expected in the intended product: heating at the formulation pH and salt level for soups and sauces; extrusion or cooking with fat and protein-network interactions for meat analogues; acidification and storage for fermented foods; and drying, moisture exposure, and rehydration for powders. Measurements should pair target-peptide retention with sensory performance and use replicate production batches to distinguish processing effects from batch variability.

### 6.7. Economic Feasibility and Production Scale

Economic feasibility should be assessed at the effective dose in the intended food, not at the water-solution threshold alone. The ingredient contribution to cost per serving equals the amount added per serving multiplied by the cost per unit mass of the standardized preparation. That amount depends on active-peptide content, recovery, and retained activity after processing. For a purified peptide, a mass balance should connect source-protein input, release, separation recovery, final purity, and dose. For a hydrolysate, batch composition and the complete sensory effect are the relevant specifications.

The 5 mg/mL solution concentration used for *Agrocybe aegerita* peptides [64] corresponds arithmetically to 5 g/L. This conversion illustrates the mass demand associated with that particular model-solution test; it is not a recommended food dose or a price estimate. Synthesis is useful for validation, but comparison with enzymatic or fermentation-based manufacture requires route-specific recovery and purification costs. Enzymes, solvent or membrane use, energy for concentration/drying, quality control, and disposal all contribute. A less purified preparation may reduce separation demands while introducing bitterness or compositional variability. The reported potency and formulation studies do not provide a common techno-economic dataset, so no commercial cost advantage can be inferred from their sensory results alone.

## 7. Challenges and Future Perspectives

### 7.1. Major Challenges in Application Translation

The central application bottleneck is obtaining enough reproducible taste-active material at a feasible dose. Low release or separation recovery can make purification impractical, even when a synthesized sequence is potent. Heating, storage, and matrix binding can change the dose needed, while bitterness and off-flavours limit the amount that can be added. Production yield should therefore be reported alongside retained activity and the usable dose, rather than optimized independently. Hydrolysates may reduce purification demands, but their composition and sensory effect must remain consistent across production batches.

Scale-up couples substrate variability with enzyme use, reaction time, separation, concentration, drying, and quality control. A route with high peptide yield may still be unattractive if the preparation requires extensive processing or exceeds the off-flavour limit at the effective dose.

### 7.2. Future Directions for Food Application

Standardization should link each sequence to its source, release or generation route, purity, taste-label definition, concentration unit, threshold criterion, panel design, and matrix composition. Model reports should disclose negative-label provenance, similarity-grouped partitions, fold-local preprocessing, configuration selection, and accessible data/code where possible. Unsuccessful candidates are needed alongside positives to evaluate prospective screening. Future prioritization should consider bitterness, stability, effective dose, and matrix compatibility together with predicted umami activity, with experiments used to resolve trade-offs rather than treating the highest umami score as the only objective.

Food trials should integrate target-peptide measurements, sensory responses, processing/storage retention, production yield, and the cost per effective serving. Safety assessment should account for source allergens, expected exposure, contaminants, and impurities introduced during production. Source familiarity does not establish the safety of every purified or modified preparation. Regulatory and labelling assessment must address the actual composition, production route, intended use, and market rather than treating natural origin or a clean-label description as authorization. Digestion studies should answer a defined exposure or safety question.

### 7.3. Conclusions

The main remaining gap is the connection between reproducible candidate selection and affordable, effective food use. Priorities are transparent taste datasets and untouched evaluation sets, prospective testing that reports unsuccessful candidates, and replicated food trials measuring effective dose, off-flavours, processing/storage losses, recovery yield, and cost per serving. Standardized reporting would make these outcomes comparable and clarify when computation reduces development effort. Safety, labelling, and consumer acceptance must be assessed for the actual preparation and intended use.

## Figures and Tables

**Table 1:** Representative food/protein sources, discovery routes, peptide candidates, reported evidence, and sequence-level evidence categories.

Food/Protein Source	Route	Peptide Candidates	Key Evidence	Evidence Category	References
Jinhua ham	LC-MS → database → ML → docking	GDR, LDLR, LEEA, LENR, LEQE, NHD, QDK, RAE, TEY, TLVE	13 candidates taste-tested; 10 retained; thresholds 0.1109–0.2634 mg/mL	E3; S, I	Cao et al. [4]
shiitake	UAEH → LC-MS/MS → Transformer → docking/MD	LPFQTE, MIPLLL, EVLPNL, EVENVI	Synthesis, sensory and e-tongue validation; threshold NR	E3; S, I	Han et al. [18]
Suanyu	Metagenome library → GAT → docking/MD	GYSSYK, LYSDSK, TRTKASY	Sensory/e-tongue; threshold 0.28 ± 0.14 mg/mL	E3; I	Ma et al. [5]
alcohol-free beer	RPLC-Q-TOF-MS → ML → docking/MM-GBSA → sensory	CTGAA, IDQILG, KDTHP, QRQ, EITGR	2081 identified; 122 predicted; 117 docked; 5 peptides tested in beer at 20 mg/L	E5	Wu et al. [28]
*Hericium erinaceus*	Sensory/peptidomics + virtual screening	WGDAP, DGFE, GGLDP, NPPGDF	Sensory, e-tongue and MSG-combination tests	E3; I	Jiang et al. [12]
fermented sausage	Metagenomics → DL ensemble/SHAP → docking/MD	DDSMAATGL, DGEEDASM, DEEEVDI	Thresholds: water 0.11/0.37/0.44; MSG 0.09/0.27/0.31 mg/mL; enhancement 18.1–27.0%	E3; S	Geng et al. [29]
soybean Source sequence: KRH47534.1 (hypothetical protein)	Virtual digestion → toxicity filter → DL → docking/MD	DSWPSL, SHHPR, LGPK, SSW	Sensory/e-tongue; SHHPR strongest electronic-tongue response	E3; I	Shen et al. [7]
grasshopper	Peptidomics → ML/virtual screening → docking/MD	GDRFL, NGWGDY, NVTY, VVNY	Threshold 0.08–0.25 mg/mL; below MSG 0.30 mg/mL	E3; S	Wu et al. [30]
porcine bone	UMPred-FRL/Umami-MRNN → docking → synthesis	LREY, HEAL, LAKVH, FQKVVA, HVKELE, AEVKKAP, KALSEEL, EAVEKPQS, KKMFETES	Threshold 0.125–0.294 mmol/L; KKMFETES lowest	E3; S	Liu et al. [20]
Pixian paste	Identification → ML exploration → docking/PTM	VEGGLR	Sensory validation; threshold 0.22 mmol/L	E3	Mei et al. [31]
Yeast extract	Sensory-guided fractionation → nano-UPLC-MS/MS → virtual screening	DWTDDVEAR and other reported candidates	13 candidates from 73 identified peptides; sensory thresholds 0.07–0.61 mmol/L	E3; S	Wang et al. [32]
Yeast extract (*S. cerevisiae*)	UHPLC-MS/MS/de novo sequencing → modelling → synthesis/sensory	FE, YDQ, FQEY, SPFSQ	Synthetic-peptide sensory confirmation; peptide-specific thresholds reported	E3; S	Gao et al. [33]
*Spirulina* biomass	Acid hydrolysis → ultrafiltration → G-25 chromatography → LC-ESI-MS/MS	Glutamyl di-/tripeptides in an umami fraction	Fraction also contains free glutamate and succinic acid; isolated-sequence activity unresolved	Fraction-level; no sequence-level E assignment	Pratama et al. [34]
Tomato proteins	Virtual digestion → ML screening → synthesis/sensory → docking/MD	AGPNY	Umami threshold 0.0625 mg/mL; sensory/e-tongue support	E3; S, I	Zhu et al. [16]

**Table 2:** Systematic comparison between wet-laboratory enzymatic hydrolysis and virtual enzymatic digestion.

Dimension	Wet-Laboratory Enzymatic Hydrolysis	Virtual Enzymatic Digestion
Candidate scale	Detected candidate count depends on preparation, fractionation, and MS coverage	Theoretical count depends on source sequences and cleavage rules; this is distinct from detected abundance
Cycle	Includes preparation, separation, identification, and experimental testing	Pre-screening includes sequence retrieval, cleavage simulation, and ranking; wet validation is additional
Cost	Requires enzymes, separation/MS, and taste testing; cost depends on workflow	Computational prescreening precedes synthesis and taste testing; total cost depends on the validation scale
Enzyme coverage	Enzymes selected and tested under defined processing conditions	Dozens of enzymes/combinations; e.g., 33 single + 528 double enzymes [15]
Main limitation	Detection and fractionation constrain the tested peptide pool; rare active components may be missed	Predicted cleavage and taste activity require separate experimental confirmation [7,15,16]
Validation requirement	Measured release and sensory activity still need sequence confirmation when single-peptide claims are made	Synthesis and identity/purity checks enable taste testing; actual release requires measurement under processing conditions; food claims require matrix testing

**Table 3:** Study-specific model evaluation and reporting audit. Predictive performance is presented separately from experimental follow-up; the table also documents negative labels, sequence separation, augmentation, and data/model accessibility. Values are not a cross-study leaderboard.

Model and Reference	Dataset and Negative Controls	Evaluation, Leakage, and Sampling	Reported Performance	Experimental Follow-Up/Interpretation	Data/Code/Model Availability
iUmami-SCM; AA/dipeptide scoring Charoenkwan et al. [48]	UMP-TR: 112 umami/241 non-umami; UMP-IND: 28/61 **Negative controls:** Bitter peptides (shared benchmark)	Internal UMP-IND holdout; selected SCM run (note c) **Sequence separation/leakage:** Exact-sequence redundancy removed **Augmentation/sampling:** Not specified	ACC 86.5%; AUC 0.898; MCC 0.679; Sn 71.4%; Sp 93.4%	Benchmark classification; not a prospective synthesis hit rate	Dataset and classifier code linked on GitHub; prediction server reported
UMPred-FRL meta-predictor Charoenkwan et al. [49]	Same UMP-TR/UMP-IND split: 112/241 train; 28/61 test **Negative controls:** Bitter peptides (shared benchmark)	Internal UMP-IND holdout; selected PF7 representation **Sequence separation/leakage:** Unique sequences reported **Augmentation/sampling:** Not specified	ACC 88.8%; AUC 0.919; MCC 0.735; Sn 78.6%; Sp 93.4%	Shared benchmark; not an external food-source cohort	Dataset download and prediction server reported; training-code availability not established here
iUP-BERT; BERT + SMOTE + SVM Jiang et al. [50]	UMP442: 112/241 train; 28/61 test **Negative controls:** Bitter peptides (shared benchmark)	Internal UMP-IND holdout; SVM, 139 BERT features, SMOTE **Sequence separation/leakage:** Unique sequences reported **Augmentation/sampling:** SMOTE; training-data use described, but within-fold order not explicit	ACC 89.9%; AUC 0.933; MCC 0.774; Sn 89.3%; Sp 90.2%	Classification metric, not experimental synthesis success	Supplementary dataset and prediction server reported; full training-code availability not established here
iUmami-DRLF; UniRep/mLSTM + LR Jiang et al. [51]	UMP442: 112/241 train; 28/61 test **Negative controls:** Bitter peptides (shared benchmark)	Internal UMP-IND holdout; LR configuration **Sequence separation/leakage:** Curated UMP442 split **Augmentation/sampling:** SMOTE evaluated; within-fold order not explicit	ACC 92.1%; AUC 0.956; MCC 0.815; Sp 96.7%	Additional literature-derived UMP-VERIFIED set: 91 positives; distinct from these metrics and prospective synthesis	Dataset URL and prediction server reported; full training-code availability not established here
Umami-Transformer Fu et al. [19]	UMP499: 212 umami + 287 non-umami **Negative controls:** Bitter peptides used as negative controls	Internal 80:20 split; 5-fold CV for development; test: 42/57 **Sequence separation/leakage:** Redundant sequences removed; homology-separated split not specified **Augmentation/sampling:** Augmentation not specified	Not tabulated; reported metric consistency unresolved (note a)	Selected short peptides synthesized and taste-tested; not a population-wide hit rate	Dataset repository linked; precise benchmark partition accessibility requires checking
UmamiMeta/ESM-MLP He et al. [44]	Collected: 972 umami + 608 non-umami; ≤15 aa: 957 + 589 **Negative controls:** Non-umami labels compiled from taste databases/literature; not one uniform assay	Internal 5-fold CV on ummpep2024; MLP_esm2 **Sequence separation/leakage:** Multiple sources combined; homology-separated classification folds not established **Augmentation/sampling:** Oversampling of each training fold described	MLP_esm2: ACC 82.0%; AUC 0.87; length-specific results vary by model	PLM-based classification; length effects and affinity calculations are separate endpoints	Dataset download, web server, and code repository reported
CNN/Transformer/LSTM/Attention ensemble Geng et al. [29]	Labelled set: 254 umami + 254 non-umami; 150,000 downstream candidates are not labelled training data **Negative controls:** Negative records from earlier predictors; no uniform negative taste assay specified	Internal 80:20 holdout; authors’ four-submodel summary (note b) **Sequence separation/leakage:** Labelled-set redundancy removal; candidate-library CD-HIT at 90% is not evidence of grouped train/test splitting **Augmentation/sampling:** Augmentation not specified	Not tabulated; summary values require clarification (note b)	Three synthetic peptides tested in water/MSG; no sausage reconstitution test	Original-data repository linked; full training-code availability not established here
Umami-BERT; BERT + Inception Zhang et al. [52]	UMP789: 275 umami + 514 non-umami **Negative controls:** Non-umami peptides sampled from pretraining data; heterogeneous bioactive peptides	Internal 5-fold CV; class ratios 1:1 and 1:2 (note d) **Sequence separation/leakage:** Homology-separated re-training folds not specified **Augmentation/sampling:** Class-ratio sampling; sample-count discrepancy remains (note d)	1:1: ACC 93.23%, MCC 0.78; 1:2: ACC 95.00%, MCC 0.85	Class-ratio-specific CV; not evidence of superior food screening	Model and data repository linked
Umami_YYDS; GBDT Cui et al. [45]	TastePeptidesDB: 483 taste peptides across taste categories; classifier task: umami/bitter **Negative controls:** Bitter peptides in an umami/bitter classification task	Calibration set for listed metrics; separate CV, holdout and sensory work also reported **Sequence separation/leakage:** Stratified split reported; homology-separated split not specified **Augmentation/sampling:** SMOTE; fold-local application not established in reviewed description	Calibration ACC 89.6%; AUC 0.98	Umami/bitter task; sensory follow-up distinct from calibration scores	Example data, code repositories, and prediction tools reported
Umami-gcForest; deep forest + ProtBERT/AAC/CTD/PAAC Ji et al. [53]	279 umami + 279 non-umami; 80:20 train/test **Negative controls:** Bitter peptides assembled from earlier model datasets	Internal 80:20 holdout used for feature-combination selection (note c) **Sequence separation/leakage:** Dataset preprocessing reported; homology-separated split not established; holdout used for feature selection **Augmentation/sampling:** Negative-class downsampling to 1:1	ACC 91.07%; F1 0.9091; MCC 0.8220; Sn 89.29%; Sp 92.86%	Separate Test_Length, Test_Novel and dual-taste evaluations reported; listed values are not those tests	Dataset/test-set repository and prediction server reported; other data on request

Table note. Predictive performance and experimental follow-up. Values belong to the evaluation setting and configuration in each row. Calibration, cross-validation, and internal holdout results are not directly comparable. A source-labelled “independent test” drawn from the same curated pool is described here as an internal holdout; separately collected external sets are identified explicitly. Experimental taste testing is not classification accuracy. Note a. Umami-Transformer: the jointly reported metrics could not be reconciled with a single standard confusion matrix for the stated 42-positive/57-negative test set. Comparable numerical performance is therefore not tabulated; the model and synthetic-peptide validation remain included. Note b. Fermented-sausage study: the source-reported four-submodel summary does not fully reproduce the arithmetic means of the submodel entries. No recalculated summary is substituted. The labelled dataset and downstream candidate library are distinct. Note c. iUmami-SCM reports a selected run rather than a run-average estimate; selection considered CV and holdout performance. Umami-gcForest used holdout scores to select feature combinations. These values are not estimates from a completely untouched external evaluation. Note d. Umami-BERT: retain separate results for 1:1 and 1:2 class ratios. The method description of UMP789 and the sample counts in the source tables are not fully reconciled; no unverified train/test counts are inferred. ACC, accuracy; AUC, area under the ROC curve; CV, cross-validation; F1, F1 score; MCC, Matthews correlation coefficient; Sn, sensitivity; Sp, specificity; SMOTE, synthetic minority oversampling technique. Class counts are umami/non-umami. NR means not reported, not zero. **Additional table note.** Data labels, leakage controls, augmentation, and accessibility reported by the source studies. Shared conditions across the first four models: the benchmark is reported as UMP-TR/UMP-IND [48,49] or the corresponding UMP442 dataset [50,51] contains 112 umami/241 non-umami peptides for training and 28/61 for testing; bitter peptides serve as non-umami controls. Homology-separated splitting is not specified. Augmentation is not specified for iUmami-SCM or UMPred-FRL. Negative controls and augmentation reporting are stated explicitly in each row; the other entries identify study-specific reporting differences. Reuse of this benchmark does not constitute four independent external validations. These reporting-audit fields summarize statements identifiable in the reviewed source reports, not a code-level reproduction. “Not specified” or “not established” indicates insufficient reporting, not proof that a procedure was absent. Exact-sequence deduplication is distinct from similarity-grouped splitting. Accessibility refers to source-reported links or statements and does not certify current server operation or full reproducibility.

## Data Availability

No new data were created or analyzed in this study. Data sharing is not applicable to this article.

## Abbreviations

AUC	area under the receiver operating characteristic curve
BERT	Bidirectional Encoder Representations from Transformers
CR	candidate-compression ratio
DL	deep learning
GMP	guanosine 5′-monophosphate
GRM1	metabotropic glutamate receptor 1
HR	experimental hit rate
IMP	inosine 5′-monophosphate
LC-MS/MS	liquid chromatography-tandem mass spectrometry
LSTM	long short-term memory
MCC	Matthews correlation coefficient
ML	machine learning
MS	mass spectrometry
MSG	monosodium glutamate
NCBI	National Centre for Biotechnology Information
T1R1/T1R3	taste receptor type 1 member 1/type 1 member 3
